# 2La Paracentric Chromosomal Inversion and Overexpressed Metabolic Genes Enhance Thermotolerance and Pyrethroid Resistance in the Major Malaria Vector *Anopheles gambiae*

**DOI:** 10.3390/biology10060518

**Published:** 2021-06-10

**Authors:** Sulaiman S. Ibrahim, Muhammad M. Mukhtar, Abdullahi Muhammad, Charles S. Wondji

**Affiliations:** 1 Vector Biology Department, Liverpool School of Tropical Medicine, Pembroke Place, Liverpool L3 5QA, UK; abdullahi.muhammad@lstmed.ac.uk (A.M.); charles.wondji@lstmed.ac.uk (C.S.W.); 2 Department of Biochemistry, Bayero University, PMB 3011, Kano 700241, Nigeria; muhammadmahemukhtar@gmail.com

**Keywords:** malaria, *Anopheles*, coluzzii, *gambiae s.s.*, 2La, chromosomal, inversion, thermotolerance, permethrin, resistance

## Abstract

**Simple Summary:**

While shifts in temperature may promote the spread of insects, exacerbating the intensity of vector-borne diseases like malaria, very high temperatures exert deleterious effects in insect vectors, forcing them to evolve/adapt genetically. These genetic adaptations may facilitate insecticide resistance through common genes involved in both processes. Here, the impact of thermal tolerance on pyrethroid resistance in the major malaria vector *Anopheles gambiae s.l.* from four localities spanning the savanna and sub-Sahel of northern Nigeria was investigated. In all four localities, *An. coluzzii* and *An. gambiae s.s*. were the only malaria vectors found. The populations were highly thermotolerant, with ~50% of mosquitoes in two sites surviving 44 °C. Thermotolerant larvae and adult mosquitoes (survivors of 44 °C) were significantly more resistant to permethrin, suggesting that prior heat-hardening facilitates insecticide resistance. Thermal tolerance and permethrin resistance were found to be associated with the 2La rearrangement (a form of chromosomal inversion). Transcriptional analysis revealed six major genes commonly overexpressed in the highly thermotolerant mosquitoes and those resistant to permethrin, suggesting common mechanisms involved in thermotolerance and insecticide resistance. These findings highlight challenges associated with insecticide-based malaria vector control, stressing the need to take environmental variables and other pleiotropic mechanisms into account for the choice of control measures.

**Abstract:**

Changes in global temperature are impacting the spread/intensity of vector-borne diseases, including malaria, and accelerating evolutionary/adaptive changes in vector species. These changes, including chromosomal inversions and overexpression and/or changes in allele frequencies of thermotolerance-associated genes, may facilitate insecticide resistance through pleiotropy. This study investigated the impact of thermotolerance on pyrethroid resistance in four populations of the malaria vector *An. gambiae s.l.,* from the savanna/sub-Sahel of northern Nigeria. *Anopheles coluzzii* and *An. gambiae s.s.* were the only malaria vectors found, sympatric in all the sites, with the former species predominant. High thermotolerance was observed, with no mortality at 38 °C, and LT_50_ of ~44 °C. Significantly high permethrin resistance was observed (mortality < 50%) in 44 °C heat-hardened (exposure to an intermediately high temperature provides protection to a more severe temperature or insecticide) larvae from two sites, BUK and Pantami, compared with the control, and heat-hardened adult females from Auyo (mortality = 3.00% ± 1.20, χ^2^ = 5.83, *p* < 0.01) compared with the control (12.00% ± 4.65). The 2La chromosomal inversion was detected at ~50% in subset of larvae and 58% in subset of adult females genotyped. A significant association was observed (OR = 7.2, *p* < 0.03) between permethrin resistance and the 2La/+^a^ rearrangement compared with 2L+^a^/+^a^, in BUK larvae. For all sites, permethrin resistance correlated with 2La/a homozygosity in adult females (R = 5.02, *p* = 0.01). qRT-PCR identified six genes commonly induced/overexpressed, including the heat shock protein 70 (AGAP004581) which was 2468× and 5× overexpressed in heat-hardened and permethrin-resistant females, respectively; trehalose-6-phosphate synthase (AGAP008227); and the ionotropic glutamate receptor genes, *IR25a* (AGAP010272) and *IR21a* (AGAP008511). This study highlights challenges associated with insecticide-based malaria vector control, and the epidemiological significance of taking climate variables into account for the design/choice of control measures.

## 1. Introduction

Weather and climate are among the drivers of *Anopheles* geographic range, intensity of transmission, and seasonality of malaria, with the burden of the disease projected to increase with climate change because of a greater geographic range of the *Anopheles* vector, shifts in phenology [1], an increase in the number of generations per year [2], a longer season, and/or an increase in the number of people at risk [3,4,5]. The populations of mosquito vectors are projected to shift, but with contrasting expansions and reductions depending on the degree of local warming and the ecology of the mosquito vectors [3], leading to regionally variable patterns [5,6] different from the current ones.

Small ectotherms (such as mosquitoes) are constrained by the extrinsic thermal environments, from microclimates to regional climates [7], and are particularly sensitive to even daily temperature fluctuations [8]. Indeed, temperature has been shown to constrain mosquito development rate, egg to adult survival and mortality rate, modify biting rate and fecundity, regulate *Plasmodium falciparum* parasite development rate and vector competence in the two major malaria vectors *Anopheles gambiae s.s.* [9] and *Anopheles stephensi* [10]. Empirical evidence from *An. stephensi* suggests a temperature optimum for transmission of 26 °C (minimum and maximum of 17 °C and 35 °C, respectively). Increasing environmental temperature during the larval stages impacts thermal performance. For example, in *An. gambiae s.s.*, an increase of 4 °C (e.g., from 23 °C to 27 °C, etc), 8 °C (27 °C to 35 °C), and 12 °C (23 °C to 35 °C) significantly increased larval mortality [11], while an increase of 8 °C significantly lowered adult survival. Another study has investigated the impact of exposure to varying temperature (40 °C, 41 °C, 42 °C, 43 °C, 44 °C, 45 °C, 46 °C, 47 °C, or 48 °C) on egg hatching of *An. gambiae s.s.* from Kenya [12], establishing that the survival of eggs was influenced by both temperature and exposure time, with 40 °C being the upper tolerable temperature, beyond which the rate of egg mortality increased linearly for any given temperature. Significant egg mortalities occurred at temperatures between 42 and 44 °C, less than 20% of eggs hatched when subjected to 45 °C for 10 min, and no eggs hatched above 45 °C. With respect to vector control, ambient temperature impacts the efficacy of many public health insecticides e.g., *An. gambiae* and *An. stephensi* [13], having a marked effect on the toxicity of the most commonly used insecticides for malaria control, stressing the need to evaluate the efficacy of insecticides/control tools under field conditions [14]. Temperature below the laboratory standard of 26 °C has been shown to increase the susceptibility of *An. stephensi* to malathion, with little impact on permethrin susceptibility [15]. An increase in temperature has been established to enhance deltamethrin resistance of a susceptible population of *An. arabiensis*, while exposure at temperatures both lower and higher than the standard insectary conditions increased mortality in susceptible *An. funestus* and resistant *An. arabiensis* [14]. 

Climate change (increased aridity and drylands) is accelerating evolutionary/adaptive changes in animal species, including insect pests and disease vectors [2,16], potentially selecting evolutionary winners, with the losers, e.g., the species lacking adaptive capacity living near physiological limits and prone to extinction [2]. These adaptive genetic changes occur at chromosomal levels (for example, chromosomal inversions [17]) and/or changes in the allele frequencies of genes involved in thermotolerance and desiccation resistance [16], with increasing periods of thermal stress and drought predicted to produce directional selection for insecticide resistance [2].

Paracentric chromosomal inversions are one of the most effective instruments for speciation and local adaptations [17,18,19], are maintained in spatially and temporally heterogenous environment, segregate along climatic gradients of aridity (https://www3.nd.edu/~nbesansk/Inversions_2018.html, accessed on 12 October 2020), and their frequencies is known to be strongly and significantly correlated with a number of adaptive, phenotypic traits in the *Anopheles*. The 2La inversion in *An. gambiae s.l*. strongly correlates with degree of aridity across environmental gradients [20,21], increasing northward from humid to the arid regions in West/Central Africa [20,22], suggesting that the 2La arrangement confers a selective advantage in xeric habitats, while the alternative 2L+^a^ arrangement is more beneficial in the mesic habitats [20]. The *An. gambiae s.l.* carrying 2L+^a^ allele have been shown to be more susceptible to *Plasmodium falciparum* infection [23]. *An. gambiae s.l.* carrying 2La allele are also associated with resistance to desiccation in adults [24,25] and thermal stress in larvae [20,26], were less prone to rest indoors [21], and it was shown that inversion 2La assorts with insecticide resistance, e.g., dieldrin plus fipronil [27].

Several studies have investigated the genetic basis of thermal stress and desiccation resistance. These include measurement of cuticle thickness and cuticular hydrocarbons (CHC) composition in 2La and 2L+^a^ karyotypes, which linked the 2La arrangement with a thicker cuticle, and differences in the CHC composition associated with lower rate of water loss for the 2L+^a^ karyotype [28]. Comparative gene expression profiling of heat hardened 2La and 2L+^a^ larvae have established a common and massive induction of a core set of heat shock genes, with the 2La allele preconditioned with a much more aggressive response, with larger numbers of upregulated genes, which are heat responsive and involved in proteolytic degradation and energy metabolism [20]. Fine-scale association mapping of desiccation tolerance within the 2La and the 2Rb inversions of *An. gambiae s.s.* has revealed dozens of significant single nucleotide polymorphisms within both the 2La and 2Rb inversions, many of which neighboured genes controlling ion channels or related functions, with transcriptional profiles strongly influenced by karyotype and genes inside rearranged regions overrepresented among those differentially expressed genome-wide [29]. Two of the top-ranking candidate genes discovered in the above recent study have prominent roles in response to environmental stimulus: AGAP006026 encodes an ionotropic glutamate receptor (IR) commonly associated with chemosensation, thermosensation, and hygrosensation [30,31] and AGAP006961 [32] encodes a heat-shock protein gene (*hsp90*).

While several studies have addressed the impact of the 2La inversion on adaptation and insecticide resistance [17,27], little is known of its the impact on pyrethroid (major ingredients in bed nets) resistance in the field population of *An. gambiae s.l.* Moreover, the few genome-wide transcriptional and association studies carried out to identify the major genes involved in thermotolerance have not investigated the pleiotropic action of the discovered genes on insecticide resistance. This constitutes a knowledge gap hampering insecticides resistance management in field populations of the major malaria vectors, within the context of globally warming world. This study hypothesized that genetic adaptation (e.g., chromosomal inversion) and overexpression of thermotolerance-associated metabolic genes are facilitating thermal acclimation and pyrethroid resistance in the major malaria vector *An. gambiae s.l.* The study investigated the thermal tolerance breadth of *An. gambiae s.l.* (*An. gambiae s.s*. and *An. coluzzii*) from four sites spanning the northern Guinea savanna and Sudan/sub-Sahel of northern Nigeria, and the role of chromosomal inversion and metabolic resistance genes in tolerance to heat stress and insecticide resistance. Thermotolerance and insecticide resistance were found to be correlated with 2La inversion polymorphism, in heterozygote (2La/+^a^) and homozygote (2La/a) forms, respectively. qRT-PCR transcriptional profiling of heat-hardened and permethrin-resistant *An. coluzzii* established the induction/overexpression of a core set of heat shock protein genes previously associated with heat stress, as well as other common heat- and pyrethroid-resistance associated genes, including two ionotropic receptors and a trehalose 6-phosphate synthase/phosphatase.

## 2. Materials and Methods

### 2.1. Sampling Sites and Mosquito Populations 

To capture heterogeneities in vector compositions and/or various chromosomal forms of the *Anopheles* collection was carried out in 4 sites spanning Guinea, Sudan, and sub-Sahel savanna of northern Nigeria (Supplementary Figure S1). Larvae collection was preferred in place of indoor-resting blood fed females to avoid bias from collection of the more endophilic species [33,34]. Collections were conducted in the rainy months of July, August, and September, 2019 (mid-July through August, to mid-September) in temporary rain puddles in (i) Bayero University Kano (BUK), situated in Sudan savanna of Kano City (11°58′17″ N, 8°35′9″ E); (ii) Gamjin Bappa, a Sudan savanna village in Karaye, Kano State (11°46′23.6′′ N, 8°00′29.9″ E); (iii) Pantami, a northern Guinea savanna town located in Gombe State (10°15′50.4″ N, 11°09′39.7″ E), and (iv) in irrigation rice paddies in Hadiyau, a village in the sub-Sahel of Auyo, Jigawa State (12°21′38″ N, 9°59′15″ E). Collections were done using classical dipping method [35] and larvae maintained under standard insectarium condition (~70–80% relative humidity and 25–27 °C), supplemented with Tetramin^TM^ Baby fish food, with 12:12 h day/night cycle. Monthly maixmum and minimum ambient temperature (https://weatherspark.com/, accessed on 6 June.2021) for the collection sites were 12 °C and 39 °C, respectively for Kano, 40 °C and 15 °C for Auyo, 38 °C and 12 °C for Gamjin Bappa, 38 °C and 14 °C for Pantami. Extended data for current monthly averages are provided in the Supplementary, File S1.

### 2.2. Morphological and Molecular Identification of Mosquito Larvae and Adults

For every collection, larvae were identified as belonging to the A*nopheles gambiae* Complex using the morphological keys [36] and maintained as described above. When required larvae were reared to adulthood, and adults maintained as above, with 10% sucrose solution. A subset of larvae and adults which were used for thermotolerance and insecticide resistance bioassays (see Section 2.3 and Section 2.4 below for details) were homogenised and DNA was extracted using LIVAK method [37]. SINE200 PCR [38] was used to identify the larvae and adults to species level.

### 2.3. Initial Assessment of Thermotolerance Profile Using Temperature Gradient

Initial thermotolerance profiling of *Anopheles* species was conducted by exposing L4 larvae to various temperature gradients (5–46 °C) for 1h, using a modified protocol of Rocca [26]. This timing was a compromise between the observation of Rocca [26] (120 min which was established as the LT_50_ at 40 °C) and 30 min at 40 °C and 43 °C which was used by Benedict et al. [39]. Larvae from BUK and Hadiyau were subject to 11 static temperature treatments (5 °C, 7 °C, 10 °C, 15 °C, 27 °C, 38 °C, 42 °C, 43 °C, 44 °C, 45 °C, and 46 °C), while those from Gamjin Bappa and Pantami were subjected to 6 temperature regimens: 38 °C, 42 °C, 43 °C, 44 °C, 45 °C, and 46 °C only (due to lack of sample). For the lower temperatures (5 °C through to 27 °C) eight replicates each of 25 larvae were used, while 4 replicates of 25 larvae were used for the higher temperatures (38 °C through to 46 °C (due to sample size)). The L4 larvae were placed in 50 mL glass tubes containing 20 mL of deionised water, pre-set in a water bath at the experimental temperatures, and allowed to remain for 1h. Tubes were cooled by transferring into water bath set to 27 °C and larvae fed with Tetramin food. Number of larvae dead at 24 h was recorded. In each assay, control groups received same treatment, except that they were maintained at 27 °C. 4 replicates of 25 L4 larvae of *An. coluzzii* (Ngoussou) were exposed to 43 °C and 44 °C, as well, to assess the thermotolerance status of a known, fully insecticide susceptible colony. The Ngoussou larvae were procured from the LITE, at LSTM, United Kingdom (https://lite.lstmed.ac.uk/, accessed on 9 December 2020).

### 2.4. Impact of Heat Hardening on Pyrethroid Resistance

To investigate the effect of short-term thermal stress [40] on pyrethroid resistance, larvae were pre-exposed for 30 min at 44 °C, with resting for 2 h at 27 °C, followed by dose-response bioassays with 12.5 mg/mL, 25, 50 and 100 mg/mL of permethrin. For each concentration, 4 replicates of 20–25 larvae were utilised in experiments which were conducted using the WHO procedure [41], with mortalities scored after 24 h. For each of the 4 concentrations, 4 replicates of 20–25 unexposed larvae were used as controls (exposed to respective concentrations of permethrin, but not 44 °C). Negative controls were also set for each treatment arms above and were (i) exposed to water containing the solvent (methanol) used to dissolve permethrin, and (ii) maintained in water alone.

To investigate impact of long-term heat-hardening on resistance, sets of the larvae which survived 44 °C were reared to adulthood and 2–5 day-old females used for WHO tube bioassays with permethrin [42]. Four replicates each of 20–25 females were exposed to impregnated papers containing discriminating doses of permethrin (0.75%) for 1 h, transferred to holding tubes and supplied with 10% sucrose. Mortality was recorded 24 h after exposure. Two controls were used: (i) 2 replicates of 20–25 adults which survived 44 °C but not exposed to insecticide; and (ii) 2 replicates of 20–25 adults unexposed to 44 °C and unexposed to insecticide.

### 2.5. Molecular Karyotyping of 2La and 2L+^a^ Inversion Polymorphism 

To establish correlation between thermotolerance and chromosomal inversion larvae were genotyped for the 2La polymorphism. These include 44 °C survivors (thermotolerant, T_R_) and those that died (T_S_). Moreover, alive (Insecticide resistant, I_R_) and dead (I_S_) larvae from 100 mg/mL permethrin exposure were also genotyped, to correlate insecticide resistance at immature stage with the inversion. In addition, adult females, permethrin-alive and permethrin-dead from WHO tube bioassays were also genotyped to establish correlation between pyrethroid resistance and inversion in adult stage. These were adults raised from larvae and they were not exposed to any heat stress (not heat-hardened). All larvae used for genotyping were identified to species level, using the LIVAK DNA extraction protocol, followed by SINE200 PCR (described in Section 2.2 above).

Molecular karyotyping was carried out using PCR [43,44] with primers 23A2 (Universal reverse), 27A2 (for 2La) and DPCross5 (for 2L+^a^). The PCR mix comprised 11.85 µL of ddH_2_0, 5 µL of 5× Buffer, 25mM MgCl_2_ (2 µL), 2.5mM dNTP mix (2 µL), 1 µM (1 µL) each of the above 3 primers and 5 U/µL of GoTaq DNA polymerase (Promega, Wisconsin, USA). Thermocycling conditions were 94 °C for 2 min, followed by 35 cycles each of 94 °C for 30 s, 60 °C for 30 s and 72 °C for 45s; and a final extension at 72 °C for 5min. PCR amplicons were separated on 2% agarose gel stained with pEqGREEN and visualised for bands. Product sizes for the 2La and 2L+^a^ arrangements were 492 bp and 207 bp, respectively, with heterozygotes having both bands.

### 2.6. Transcriptional Profiling of Thermotolerance-Related Genes Using qRT-PCR 

To investigate the potential role of pleiotropic genes on thermotolerance and insecticide resistance, 3–4 day-old females [40] which survived 44 °C exposure for 1 h (and allowed to rest for 2 h [45]), those which survived exposure to 0.75% permethrin and unexposed females were used for qRT-PCR, targeting 9 genes previously associated with thermotolerance and/or insecticide resistance [20,31,46,47,48,49]. These include 6 heat shock protein genes: [*hsp90* molecular chaperone HtpG (AGAP006959), *hsp90* beta (AGAP001424), *hsp83* (AGAP006958), *hsp70* 1/8 (AGAP004944), *hsp90* ATPase activator (AGAP010514) and *hsp70* (AGAP004581)]; the trehalose-6-phosphate synthase/phosphatase (*TPS 1/2*, AGAP008227), and the 2 ionotropic receptor genes, *IR21a* (AGAP008511) and *IR25a* (AGAP010272). The primers utilised for the qPCR are provided in the Supplementary Table S1. 

The qRT-PCR was carried out using cDNA extracted from 1 µg of total RNA from three biological replicates each of the female survivors of heat hardening at 44 °C (HH_R_), females unexposed to temperature stress, Control (UNX_C_), females which survived exposure to 0.75% permethrin (Insecticide resistant, I_R_) and unexposed females from the fully insecticide susceptible laboratory colony, Ngoussou. Protocol followed was as done previously [50], with relative expression level and fold change (FC) of each gene in exposed and control females relative to susceptible calculated according to the 2^−ΔΔCT^ method, incorporating the PCR efficiency [51], after normalization with the housekeeping genes, ribosomal protein S7, *RSP7* (AGAP010592) and elongation factor Tu (AGAP005128).

### 2.7. Data Analysis 

R version 3.6.1 (https://cran.r-project.org/bin/windows/base/, accessed on 31 January 2021) was utilized to calculate Odds Ratio (epiR package), to establish the relationship between thermotolerance and permethrin resistance with the 2La inversion polymorphism. LT_50_ and LC_50_ were calculated and dose-response plots created with generalised linear model (glm) using the MASS package. Plots of results of larval and adult bioassays were made using the GraphPad Prism version 7.02 (GraphPad Inc., La Jolla, CA, USA). Statistical analyses were carried out using a two-tailed Chi-Square test of independence as implemented in GraphPad Prism. The 2La genotyping data from both larvae and adults were also analysed using multiple correspondence analyses with FactoMineR (for analysis) and factoextra (ggplot2-based visualisation) packages of R, to establish a correlation between phenotype and genotype.

## 3. Results

### 3.1. Distribution and Composition of Anopheles Gambiae Species 

The larvae found in all the 4 sites, in collections spanning 3 months were *An. coluzzii* and *An. gambiae s.s.* However, these species varied in their composition, with *An. coluzzii* predominant in Auyo and BUK (Table 1, Supplementary File S3) while the two species were on average equally distributed in Gamjin Bappa and Pantami.

### 3.2. Thermotolerance Profile and Its Intensity in Anopheles Gambiae s.l. Larvae

**Figure 1:**
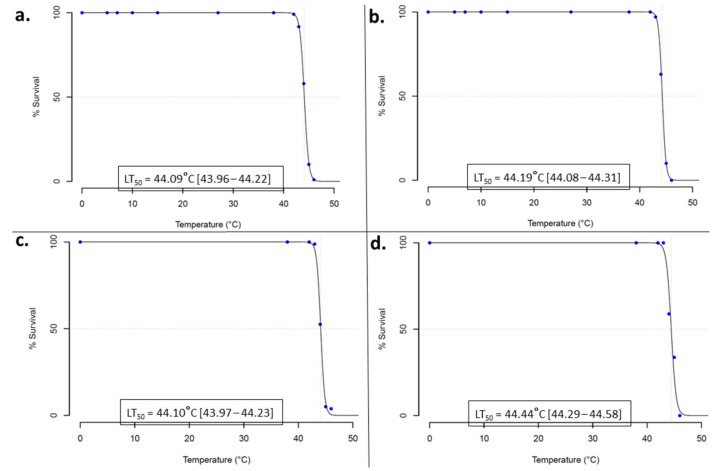
Thermotolerance profile of various populations of *Anopheles gambiae s.l.* larvae. Dose-response (temperature-course) bioassays for (**a**). Auyo, (**b**). BUK, (**c**). Gamjin Bappa and (**d**). Pantami. LT_50_, the temperature that killed 50% of the larvae are given inside rectangular inset with 95% confidence intervals in square brackets.

Initial exposure to various temperatures revealed a high thermotolerance, with no mortality in the larvae which were exposed to lower temperature (5–38 °C) (Supplementary File S1). For Auyo populations mortalities of 1%, 9%, 42%, 90%, and 99% were obtained at 42 °C, 43 °C, 44 °C, 45°C and 46 °C respectively, resulting in estimated LT_50_ (lethal temperature that killed 50% of the larvae) of 44.09 °C (95% CI: 43.96–44.22) (Figure 1). Similar pattern was observed in the BUK population with no mortality at 42 °C, but mortalities of 3.5%, 38%, 90%, and 100% at 43, 44, 45, and 46 °C, respectively (LT_50_ = 44.19 °C, CI: 44.08–44.31). No mortality was also obtained at 42 °C with larvae from Gamjin Bappa, but at 43 °C, 44 °C, 44 °C and 46 °C mortality increased to 1.25%, 47.5%, 95%, and 96.25%, respectively (LT_50_ = 44.10 °C, CI: 43.97–44.23). For Pantami, no mortality was seen at 42 °C and 43 °C, but at 44 °C, 45 °C and 46 °C, 42.5%, 66.25%, and 100% of the larvae died (LT_50_ = 44.44°C, CI: 44.29–44.58). Highest mortality was obtained from the Ngoussou, 37% for 43 °C and 100% for 44 °C, suggesting that the laboratory colony is more thermo-susceptible than the field populations. 

### 3.3. Impact of Heat Hardening on Pyrethroid Resistance

**Figure 2:**
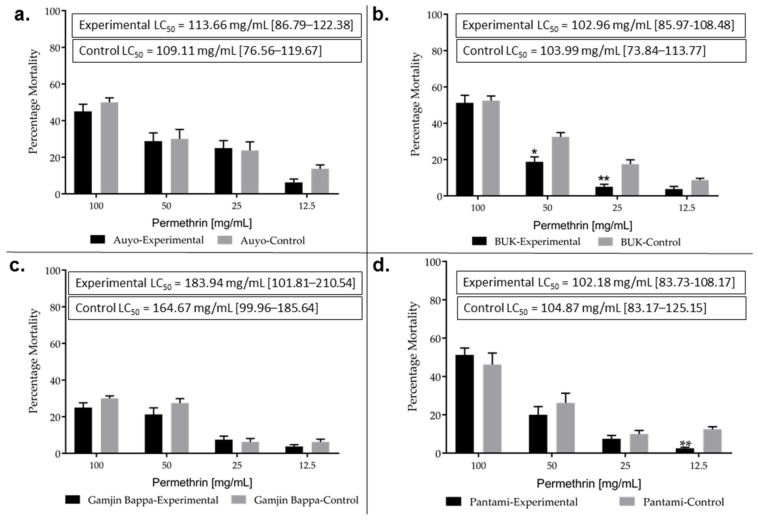
Resistance profiles of *Anopheles gambiae s.l.* larvae. Results of WHO larval bioassay with permethrin, for (**a**). Auyo, (**b**). BUK, (**c**). Gamjin Bappa and (**d**). Pantami. * and ** = statistically significant at *p* < 0.05 and *p* < 0.01, respectively from two tailed χ^2^ square test of independence. LC_50_ for experimental and control larvae for each of the 4 sites are provided in rectangular inset with 95% confidence limits.

Bioassay using the 44 °C heat-hardened larvae revealed a high permethrin resistance in all the four populations, with mortalities of <50% on average for even 100 mg/mL concentration (Figure 2, Supplementary File S2). Mortalities followed dose-dependent pattern with lowest obtained from 12.5 mg/mL permethrin. Significant differences in the mortalities were observed between the experimental and control larvae, for BUK (50 mg/mL, χ^2^ = 3.908, *p* < 0.05 and 12.5 mg/mL, χ^2^ = 6.25, *p* < 0.01) and for Pantami (12.5 mg/mL, χ^2^ = 5.76, *p* < 0.01) (Figure 2b,d, respectively). However, sample sizes (~80 larvae for this concentration) from each of these two sites could have impacted this outcome. The LC_50_ (concentration of permethrin that killed 50% of the larvae) was high (in ranges of 102–184 mg/mL), with Gamjin Bappa exhibiting the highest LC_50_, and specifically the experimental larvae of this population exhibiting higher LC_50_ compared with the control.

**Figure 3:**
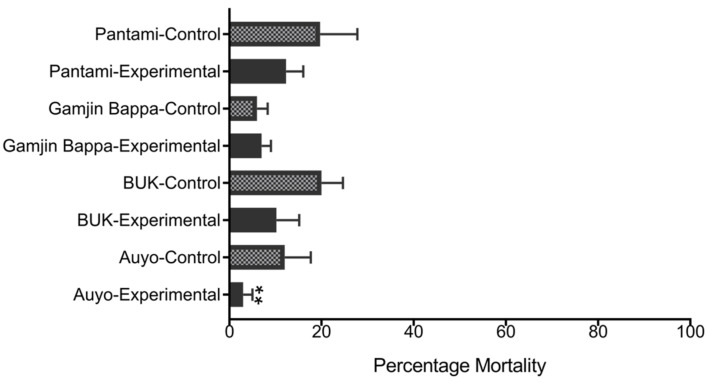
Result of susceptibility bioassay using heat-hardened and control adult *An. gambiae s.l.* females. WHO bioassays with 0.75% permethrin. ** = significantly different from the control at *p* < 0.01.

Adult bioassay with permethrin revealed high resistance, with mortalities of less than 20% in both experimental and control cohorts. However, differences in mortalities were observed between the experimental cohorts (raised from larvae which survived exposure at 44 °C) and control (Figure 3). For example, significantly lower mortality (3.00% ± 1.20, χ^2^ = 5.83, *p* < 0.01) was obtained from Auyo experimental cohort compared with control (12.00% ± 4.65). For the other three sites, mortalities were not significantly different between experimental and control, though in all cases it was higher in the control.

### 3.4. Role of 2La Chromosomal Inversion on Thermotolerance and Pyrethroid Resistance

To investigate the role of chromosomal inversion on thermal stress and permethrin resistance, larvae used for thermotolerance tests (alive and dead from 44 °C exposure), and those exposed to 100 mg/mL permethrin were karyotyped for the 2La inversion (Supplementary Figures S2 and S3, respectively for agarose gel pictures). Moreover, adults exposed to 0.75% permethrin (both alive and dead) were genotyped for the inversion (Supplementary Figure S4). 

**Figure 4:**
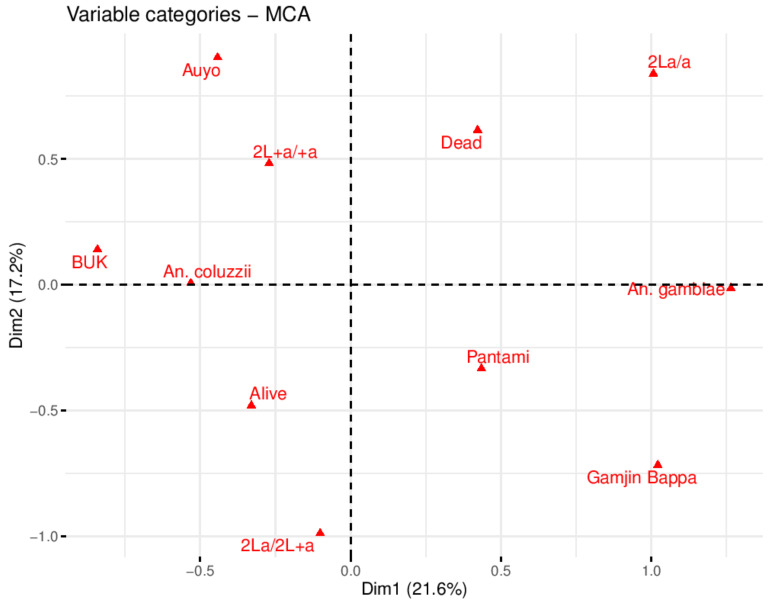
MCA plot of variables from larvae exposed to 44 °C heat stress. Asymmetric/biplot variables on dimensions 1 and 2. Variable categories with similar profiles are grouped together. Negatively correlated variable categories are positioned on the opposite side of the plot origin (opposed quadrants). Variables cluster close to the pole, positive or negative of each dimension indicating how important is their contribution to the dimension.

For BUK larvae exposed to 44 °C, significant association (odds ratio, OR = 7.2 (1.08 − 4.79), χ^2^ = 4.68, *p* < 0.03)) was observed between survival and heterozygosity (2La/+^a^ arrangement) compared with 2L+^a^/+^a^ form (Supplementary Table S2). For the other three sites, the differences were not significant, as well as for the combined data. Overall, from the larvae successfully genotyped, 55 (56.12%) were alive and 43 (43.88) dead, and the distribution of 2La/a, 2La/+^a^ and 2L+^a^/+^a^ were 16 (13.33%), 36 (36.73%) and 46 (46.94%), respectively. Multiple correspondence analysis (MCA) identified variables that explained highest variability in the data. The eigenvalues (which determine the number of variables to consider) revealed dimensions 1–5 as constituting 81.8% in the variability in the data (Scree plot in Supplementary Figure S5a showed the contribution of the dimensions, and S5b and S5c describe the variable contributions and cos2 (inertia of the variables), respectively). Variable categories with similar profiles clustered together on the factor map, for example, Pantami, Gamjin Bappa, and *An. gambiae s.s*. clustered on the positive quadrant of dimension one consistent with the high percentage of this species in the above two sites (Figure 4). The alive and 2La/+^a^ clustered together on the negative quadrant of dimension 2 supporting the significant correlation observed between 2La/+^a^ and heat stress survival. Auyo, BUK, *An. coluzzii* and 2L+^a^/+^a^ clustered together since this species is the dominant one in these two sites, and since 2L+^a^/+^a^ arrangement is not linked to thermal and desiccation tolerance, an alternative mechanism is possibly contributing to the thermotolerance in *An. coluzzii* (note that the distance between alive and *An. coluzzii* indicates their similarity compared to the variable dead, which cluster with 2La/a arrangement). The variables *An. coluzzii* and *An. gambiae s.s.* which clustered on the opposite side of dimension 2 pole contributed the most to the inertia of the factor map, followed by 2La/+^a^ inversion arrangement (Supplementary Figure S6).

For larval bioassay with permethrin, no significant difference in distribution of 2La inversion was observed between alive and dead in each site and for the combined data from all the four sites. From the larvae successfully genotyped, 41 were resistant (48.8% alive) and 43 were dead (51.2%), and the distribution of 2La/a, 2La/+^a^, and 2L+^a^/+^a^ was 15 (17.85%), 32 (38.09%), and 37 (44.04%), respectively.

**Figure 5:**
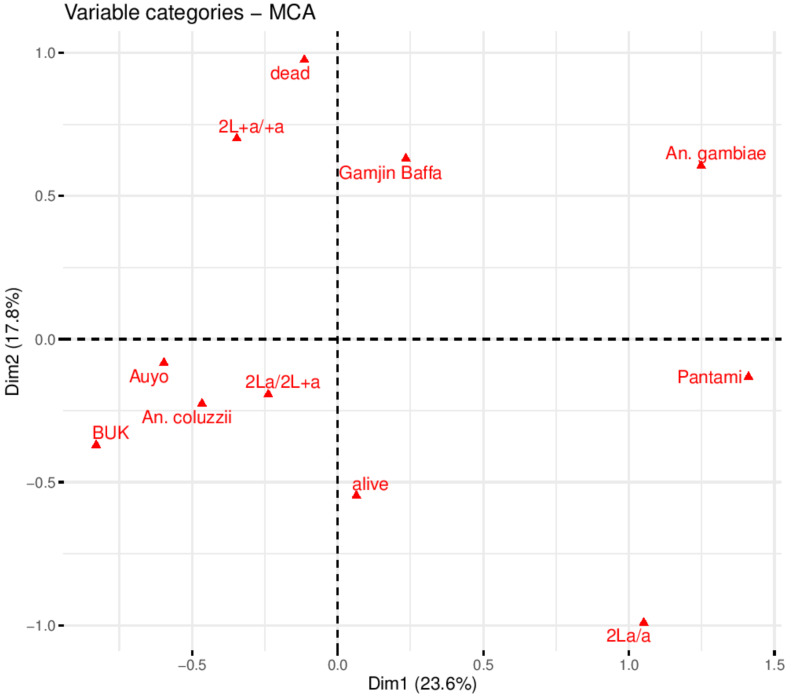
MCA plot of variables from female adults exposed to permethrin. Asymmetric/biplot of variables on dimensions 1 and 2. Variable categories with similar profiles are grouped together. Negatively correlated variable categories are positioned on the opposite side of the plot origin (opposed quadrants). Variables cluster close to the pole, positive or negative of each dimension indicating how important is their contribution to the dimension.

In contrast, for the adult bioassays there was low frequency of 2La/a arrangement among the dead females, with none in BUK and Gamjin Bappa. A significant association (OR = 5.02 (1.48–6.93), χ^2^ = 7.45, *p* = 0.01) was observed between survival and 2La/a homozygosity when alive and dead females were compared, for all data. Overall, from the adults successfully genotyped, 83 (72.8%) were alive and 31 were dead (overall percentage mortality = 27.2), and the distribution of 2La/a, 2La/+^a^, and 2L+^a^/+^a^ was 25 (21.92%), 42 (36.84%), and 47 (41.23%), respectively. The eigenvalues revealed dimensions 1–5 as contributing 84.8% in the variability of the data (Scree plot in Supplementary Figure S5d shows contribution of dimensions, and S5e and S5f demonstrated the variable contributions and cos2 (inertia of the variables), respectively). Variables 2L+^a^/+^a^ and dead clustered together (Figure 5); Auyo, BUK, *An. coluzzii*, and 2La/+^a^ clustered together, consistent with the high percentage of this species in these sites. Alive and 2La/a were most close in the right quadrant of dimension 1, supporting the observation of a significant association between permethrin survival and the inversion in homozygous state. As in the thermotolerance bioassay biplot, the variable category species (*An. coluzzii* and *An. gambiae s.s*.) contributed the most inertia for the dimensions (Supplementary Figure S7).

### 3.5. Impact of Heat Hardening on Expression Profile of Thermotolerance- and Resistance-Associated Genes

**Figure 6:**
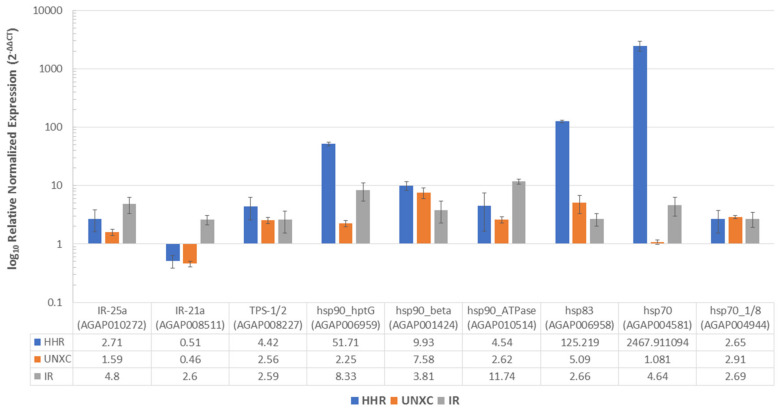
Differential expression profiles of 9 genes putatively linked with thermotolerance and/or insecticide resistance. Comparison of heat-hardened adult, female *An. coluzzii* (HHR), Unexposed females (UNXC) and adult survivors of permethrin exposure (IR) to the fully susceptible laboratory colony, Ngoussou. Fold-changes were obtained from the average of 3 independent biological replicates, each of 3 technical replicates. Error bars represent standard deviation. Fold change data table shown below the x axis. Y-axis log to base 10 transformed due to the very high overexpression of *hsp70* in HHR females.

Comparative profiling of expression of nine genes associated with thermotolerance and/or resistance revealed the common metabolic genes differentially expressed with respect to thermal stress and pyrethroid exposure. Three major heat shock protein genes were highly overexpressed in heat-hardened mosquitoes and to some level in permethrin resistant females (Figure 6). The most overexpressed gene was *hsp70* (AGAP004581), massively induced in heat hardened (HHR) females, with a fold change (FC) of 2468, FC of 4.64 in permethrin-resistant females (IR), and FC of 1.08 in unexposed females (UNXC), all compared to Ngoussou. This was followed by *hsp83* (AGAP006958) with FC of 125.2, 5.9, and 2.7 in HHR, UNXC, and IR, respectively. The third most overexpressed gene was the *hsp90* hptG (AGAP006959) which was also induced in HHR and IR, with FC of 51.7 and 8.3, respectively. The *TPS-1/2* (AGAP0008227) was also induced, with the highest FC of 4.42 in HHR, and comparable expression of 2.59 and 2.56 in the IR and UNXC, respectively, highlighting its constitutive overexpression in the field populations. The two ionotropic receptor genes were also differentially expressed with *IR-25a* (AGAP010272) having the higher FC of 4.8 in IR, followed by HHR (FC = 2.71) and UNX (1.59). The *IR-21a* (AGAP008511) was only induced significantly in the permethrin resistant (IR) females (FC = 2.6), suggesting the preeminent role of this gene in insecticide resistance.

## 4. Discussion

Under the projection of the future warmed world, mosquito species equipped with genetic and/or plastic advantages may better survive harsh conditions of tropical Africa, particularly the xeric, arid Sahel in the West Africa. Chromosomal inversion polymorphism (which promote ecological flexibility and enable exploitation of heterogeneous environments) and/or a suite of pleiotropic genes involved in thermosensation/thermoregulation, hygrosensation and chemosensation conferring adaptive benefit in arid region, as well as protection from insecticide-based vector control measures may select for a DVS in the future. Understanding the role of inversion polymorphism and the genes enhancing life traits in the major malaria vectors will help in reducing malaria risk and promote evidence-based resistance management.

### 4.1. Evidence of Sympatry between An. coluzzii and An. gambiae s.s. in Northern Nigeria

In this study the two malaria vectors, *An. coluzzii* and *An. gambiae s.s*. were the only species found in four sites spanning northern-Guinea savannah, Sudan savanna and sub-Sahel of northern Nigeria. Despite the sympatric existence of these sibling species no *An. coluzzii*/*An. gambiae s.s.* hybrids were obtained strengthening the evidence of positive assortative mating within these molecular forms [22]. However, in line with our recent findings across Sudan/Sahel [33,34,35,52,53], *An. coluzzii* was the major vector found in all the sites, even though *An. gambiae s.s.* was also in high proportion in the less xeric Gamjin Bappa and Pantami. Previous observations have described *An. arabiensis* as the dominant vector species (DVS) in the northern-most drier/arid environments of Sudan Savannah and Sahel, extending along increasing latitudinal cline, for example, in Niger, Nigeria and Cameroon [22,54,55,56]. However, within the last few years, the pervasive *An. coluzzii* has become the DVS in the Sahel of southern Niger and central Chad, and in the sub-Sahel of northern Nigeria and Cameroon. This is probably due to this species higher exploitation of breeding sites associated with anthropogenic activities and behavioural plasticity to avoid predators [57], and surviving a long dry season in situ/aestivation, which allows it to predominate and become the primary force of malaria transmission [58,59]. Moreover, photoperiod and lower nightly temperature have been shown to significantly increase the longevity of the *An. coluzzii*-a mechanism which will allow it to diapause in the dry season and re-establish first in the early rainy season [60].

### 4.2. Evidence of High Intensity Thermal Stress Tolerance in Anopheles gambiae s.l. Larvae

The larvae used in this study exhibited a broad thermal breadth with no mortalities between 5–38 °C. The high thermotolerance observed in these populations (LT_50_ of ~44 °C) suggests that the larvae are equipped with mechanisms to survive in xeric conditions. A study on the thermal tolerance/egg hatching ability of *An. gambiae s.s*. has established upper tolerable temperature of 40 °C, with mortalities increasing linearly with time above this temperature, reaching 100% at 44 °C for 30 min, and with no egg hatching for 10 min exposure at 45 °C [12]. This pattern is similar to the observation of our study. Protein denaturation/degradation has been shown to occur in temperatures between 42 and 45 °C [12,61]. Another study carried out two decades ago have utilised ranges of temperature (37–44.5 °C) to establish in *An. albimanus* larval mortality occurring in a very narrow temperature range, between 40 and 43 °C [39], with 100% of larvae dead at 44.5 °C. The findings of the present study closely agree with the observations of the above study. Another study on *An. gambiae s.s.* have used 40 °C to estimate relationship between exposure time and survival rate, showing that mortality increased linearly with time [26]. Moreover, increased temperatures (23–35 °C for rearing to adulthood) have been shown to decrease *An. gambiae* larvae survival and reduce adult longevity [10].

### 4.3. Heat Hardening Enhances Insecticides Tolerance in Larvae and Adult Anopheles Gambiae s.l.

Temperature has been shown to be a critical factor modulating insecticide resistance. In this study short-term exposure at 44 °C enhanced permethrin resistance in both larvae and adults *An. gambiae s.l.* A previous study has reported augmentation of pyrethroid resistance in both resistant and susceptible populations of adult *An. arabiensis* exposed to 37 °C and 39 °C [40], with the insecticide resistant population living longer at higher temperature. In contrast, another recent study has found that increasing temperature enhanced the deltamethrin resistance of the susceptible *An. arabiensis* populations while exposure at temperatures both lower and higher than standard insectary conditions increased mortality in resistant population of this species [14]. Other studies have shown a positive temperature coefficient on insecticides, e.g., in *An. gambiae* and *An. stephensi* [13], and in other non-Anopheles insects [62].

### 4.4. 2La Chromosomal Inversion Enhances Thermotolerance and Permethrin Resistance

Chromosomal inversion is known to segregate along climatic gradient of aridity, increasing in frequency along geographical clines, from mesic to xeric environments of the arid regions [21,24]. In this study, frequency of the 2La arrangement was found to be on average between 50% in the larvae karyotyped and 58% in the adults, with the heterozygote arrangement (2La/+^a^) on average twice the frequency of homozygote form. The 2La homozygotes have been shown to be absent in cyclodiene resistant populations of *An. gambiae s.s.* from northern Nigeria [27]. Moreover, the frequency of 2La inversion in natural populations of *An. gambiae* from Kenya has been shown to decrease from 100% to 17% in less than a decade due to pressure from increased ownership and use of insecticide treated nets which could select against house entering or indoor resting [63], reversing the selection of 2La chromosomal form.

This study established a correlation between 2La inversion polymorphism (heterozygote form, 2La/+^a^ only) and thermotolerance in a single population out of the four studied. However, further genotyping needs to be carried out with larger sample to confirm this pattern. The finding of high frequency of heterozygote arrangement 2La/+^a^ suggests its possible association with phenotypic advantage, keeping adaptive alleles in heterozygote state, to avoid the otherwise highly deleterious homozygote alleles [64]. Adult *An. gambiae s.s.* carrying 2La allele are known to be desiccation resistant [24,25] and heat-hardened larvae carrying the arrangement exhibited thermal stress tolerance higher than alternative arrangement [20,26]. 

Considering the data from all the four sites, the 2La/a arrangement was also found associated with permethrin resistance in adults, when compared to 2L+^a^/+^a^. Inversion in chromosome 2 have been shown to modulate insecticide resistance in *Anopheles* mosquitoes. For example, 2Rb inversion was associated with DDT resistance in *An. arabiensis* from Ethiopia [65] and *An. gambiae* from northern Nigeria [66], and 2La/+^a^ heterozygote *An. gambiae* has been shown to be resistant to cyclodienes [27]. The link between inversions in chromosome 2 and insecticide resistance is not surprising since many detoxification genes are located within the inverted regions in this chromosome, e.g., the voltage-gated sodium channel gene, *CYP6P3* and *CYP6P4* which are located within the 2La and 2Rc inversions, respectively [67].

### 4.5. Common Metabolic Resistance Genes Are Associated with Thermotolerance and Permethrin Resistance

Short term heat-hardening has been shown to benefit *Drosophila* [68,69] by inducing heat shock proteins [45] which act as molecular chaperones for denatured proteins from heat stress [70]. In this study, short-term heat-hardening induced overexpression of several heat shock protein genes, most especially the *hsp70* (AGAP004581). This inducible gene has been implicated in thermal acclimation in *Drosophila*, with level of its expression increasing with time of exposure [45] and has been known to be involved in ameliorating the stress of blood-feeding, which is associated with elevated body temperature in *Aedes aegypti* [47]. The fact that this chaperone protein is also overexpressed in permethrin-resistant females suggest its pleiotropic effect toward insecticide resistance. Three other hsp genes found to be overexpressed in both heat-hardened and permethrin-resistant cohort are heat shock protein genes *hsp90* (AGAP006959, hptG), AGAP006958 (part of the three tandemly arranged *hsp83* genes) and *hsp90* co-chaperone Aha1. These three hsp genes together with the *hsp70* were among the core set of hsp genes involved in a common and immediate response to thermal stress in *An. gambiae* populations [20]. The other two hsp genes found to be overexpressed in the natural populations compared with Ngoussou were *hsp90_*beta and *hsp70* 1/8.

Trehalose (synthesized by *TPS 1/2*) contribute to the maintenance of warm temperature, a condition needed for survival of *Anopheles* [46,71] and is known to promote longevity, fecundity and cold tolerance in insects [49], as well as stabilizing proteins during thermal stress [72]. *TPS 1/2* was also found to be overexpressed in heat-hardened, unexposed, and permethrin-resistant females, compared with Ngoussou, with the highest expression in heat-hardened females. Ionotrophic receptors have been shown to be a requirement for humidity sensing (hygrosensation), with *IR25a* and *IR21a* known to mediate both humidity and temperature preference in the fruit fly, *Drosophila melanogaster* [31,48], in addition to *IR21a* driving heat seeking and heat-stimulated blood feeding in *An. gambiae* [48]. The level of expression of *IR25a* was found to be higher than *IR21a*, with the highest expression in permethrin-resistant individuals, followed by heat-hardened cohorts. This suggests a possible role of these glutamate receptors in thermotolerance and insecticide resistance.

Amongst the genes found to be induced/overexpressed, *hsp70* (AGAP004581), AGAP001424 (*hsp90*), AGAP004944 (*hsp70* 1/8), *TPS 1/2,* and *IR25a* are among the set of genes significantly upregulated from a recent RNAseq-based genome-wide transcriptional analysis in pyrethroid resistant populations of *An. coluzzii* from the Sahel (in preparation). Additional work needs to be done to fully elucidate the molecular basis of thermotolerance and its link to insecticide resistance. For example, the role of heat shock proteins in chaperoning the major insecticide resistance genes (using functional genomics and protein biochemistry) can be exploited as Achille’s heels to target insecticide resistant mosquitoes. 

## 5. Conclusions

Temperature critically influences the life traits of insects, and its increase lead to more generations per year, increase species range, and favour the survival of insecticide resistant *Anopheles* vectors, impacting epidemiological effects in terms of malaria transmission. This study has established sympatric existence of the major malaria vectors *An. coluzzii* and *An. gambiae s.s.* in northern Nigeria and shown that the species are highly thermotolerant and highly resistant to permethrin, in both immature and adult stage. The preliminary findings of this study suggest the role of 2La chromosomal inversion in both thermotolerance (in larvae) and permethrin resistance (in adults). Common metabolic resistance genes are potentially involved in thermotolerance and permethrin resistance in adult *An. coluzzii*, the DVS in the localities sampled. These findings highlight the challenges associated with malaria vector control and provide a glimpse of the mechanisms that mosquitoes are likely to use to adapt to future temperature extremes and the sustained usage of insecticide-based malaria control tools.

## Figures and Tables

**Table 1:** Distribution and composition of *An. gambiae s.l.* species.

Sampling Site	44 °C (Larvae)		100 mg/mL Permethrin (Larvae)		0.75% Permethrin (Adults)	
	*An. coluzzii*	*An. gambiae s.s.*	*An. coluzzii*	*An. gambiae s.s.*	*An. coluzzii*	*An. gambiae s.s.*
Auyo	19	4	14	3	25	3
BUK	25	2	18	2	31	1
G/Bappa	8	11	10	7	17	11
Pantami	18	12	16	14	10	16
Sub-total	69 (70.4%)	29 (29.6%)	58 (69%)	26 (31%)	83 (72.8%)	31 (27.2%)
Total	98		84		114	

## Data Availability

The raw data generated from this study are available in the supplementary files.

## Supplementary Materials

The following are available on https://www.mdpi.com/article/10.3390/biology10060518/s1, Figure S1: Map of the four sampling sites in northern Nigeria. Red star for BUK and Gamjin Bappa (Kano State), Auyo (Jigawa State) and Pantami (Gombe State); Figure S2: Agarose gel of 2La karyotyping using larvae from heat stress bioassay. a. top panel is for Auyo alive (lanes 1–13) while the lower panel is Auyo dead (lanes 1–9); b. BUK alive 1–13; c. BUK dead, 1–15; d. Gamjin Bappa alive (1–11) and dead (12 and 13); and e. Gamjin Bappa dead (1–3), Pantami alive (4–19) and Pantami dead (20–33); Figure S3: Agarose gel of 2La karyotyping using larvae from permethrin bioassay. a. BUK alive (lanes 1–9), BUK dead (10–20), Gamjin Bappa alive (21–26) and Gamjin Bappa dead (27–37); b. Pantami alive (1–15) and Pantami dead (16–30); c. Auyo alive 1–11 and Auyo dead 12–17. Number 18 not identified from species identification; Figure S4: Agarose gel of 2La karyotyping using adult females from permethrin bioassay. a. Auyo alive (lanes 1–16), Auyo dead (17-28), BUK alive (29–31); b. BUK alive (1–19), BUK dead (20–29), Pantami alive (30); c. Pantami alive (1–16), Pantami dead (17–26), Gamjin Bappa dead (27–35); and d. Gamjin Bappa alive (1–19); Figure S5: Relative contribution of variables on the factor map. a., b. and c. are Scree plot of the 7 dimensions, cos2 and variables contributions, respectively for dimensions 1–5, from heat stress bioassay. Highest values reflect highest contribution to the dimension (variability); d., e. and f. are Scree plot of the 7 dimensions, cos2 and variables contributions, respectively for dimensions 1-5, from adult bioassays with permethrin; Figure S6: cos2 of variables with *An. coluzzii* and *An. gambiae s.s.* (*An. gambiae* in the plot) exhibiting the highest inertia/contribution; Figure S7: cos2 of the variables with *An. coluzzii* and *An. gambiae s.s.* (*An. gambiae* in the plot) exhibiting the highest inertia/contribution; Table S1: Primers used for qRT-PCR; Table S2: Summary of the 2La inversion distribution in larvae and adult *Anopheles* mosquitoes ; File S1: Results-Temperature-Gradient-and-LT50s, File S2: Results-Larval-and-Adult-Bioassays-Rowdata, File S3: Results-Inversion-Polymorophism-Genotyping-Rowdata.
