# Supplementary material for: 2La Paracentric Chromosomal Inversion and Overexpressed Metabolic Genes Enhance Thermotolerance and Pyrethroid Resistance in the Major Malaria Vector Anopheles gambiae
Source: Biology (Basel). 2021 Jun 10;10(6):518. doi: 10.3390/biology10060518 (PMC8230517; doi:10.3390/biology10060518)
Supplement: Supplementary file 1 [file biology-10-00518-s001.zip › biology-1178459-supplementary for conversion.pdf]

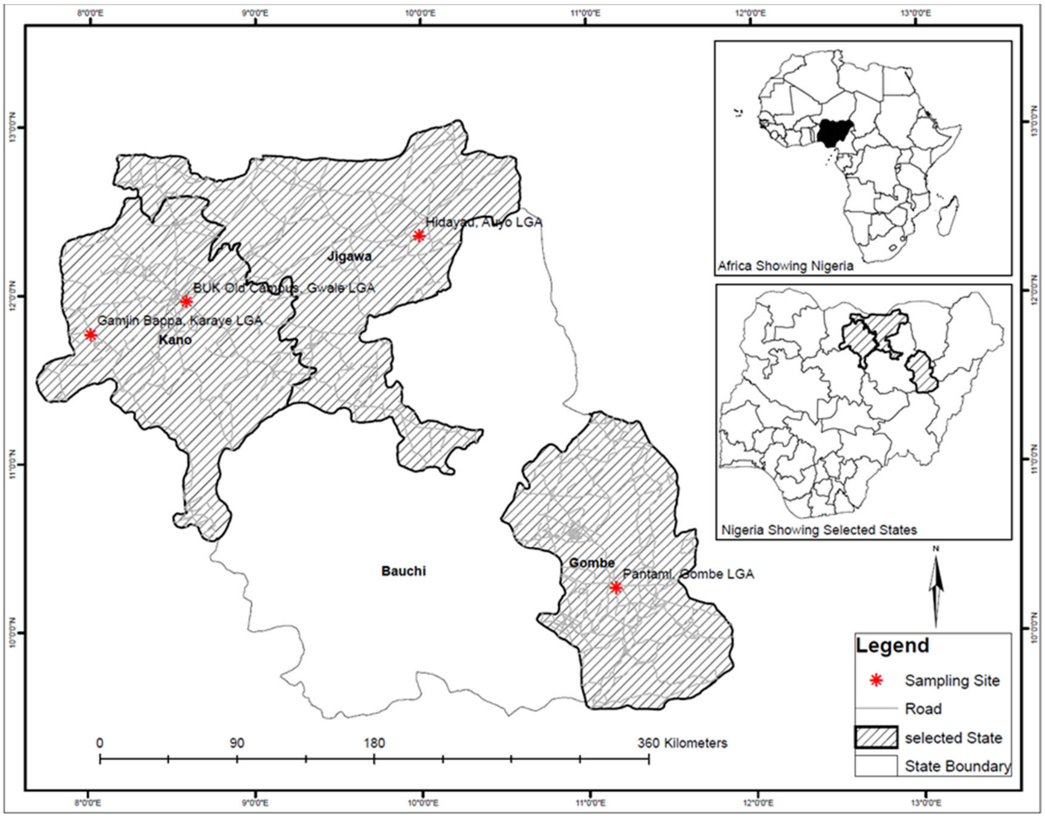

Figure S1. Map of the four sampling sites in northern Nigeria. Red star for BUK and Gamjin Bappa (Kano State), Auyo (Jigawa State) and Pantami (Gombe State).

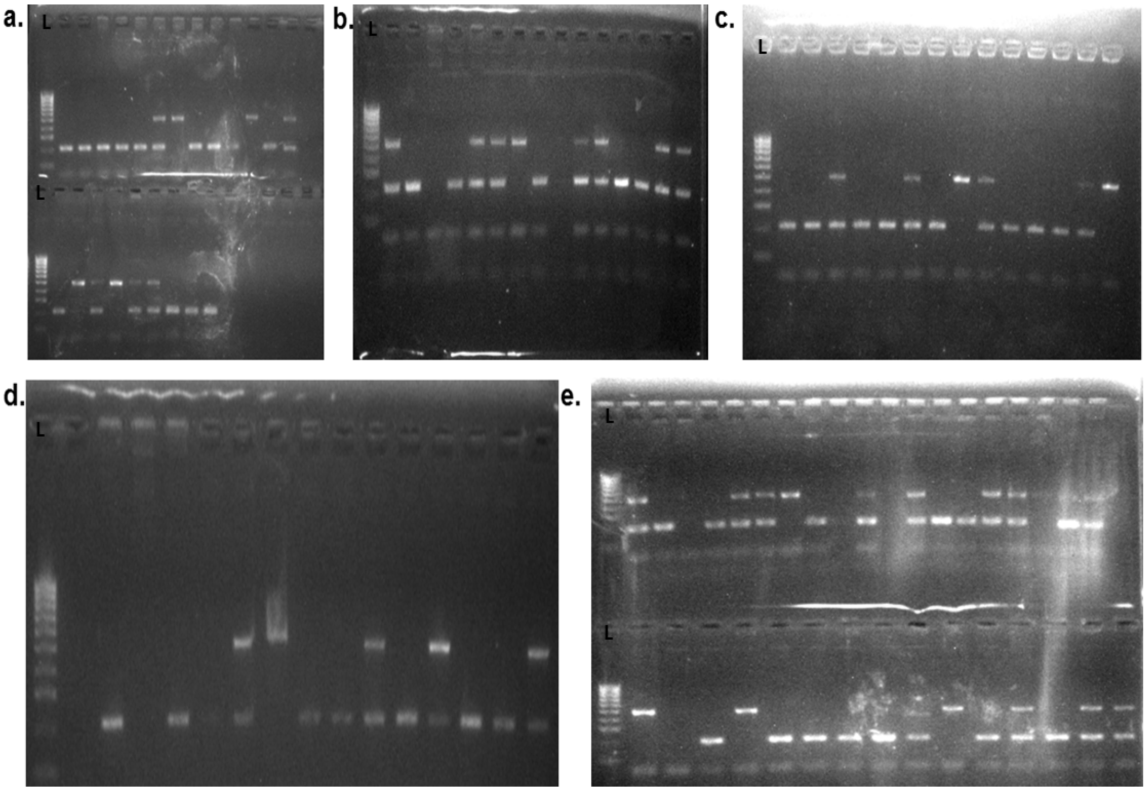

Figure S2. Agarose gel of 2La karyotyping using larvae from heat stress bioassay. **a**. top panel is for Auyo alive (lanes 1–13) while the lower panel is Auyo dead (lanes 1–9); **b.** BUK alive 1–13; **c.** BUK dead, 1–15; **d.** Gamjin Bappa alive (1–11) and dead (12 and 13); and **e**. Gamjin Bappa dead (1–3), Pantami alive (4–19) and Pantami dead (20–33). L is Bioline 100 bp DNA ladder ((100–1013 bp (40–200 ng/band)).

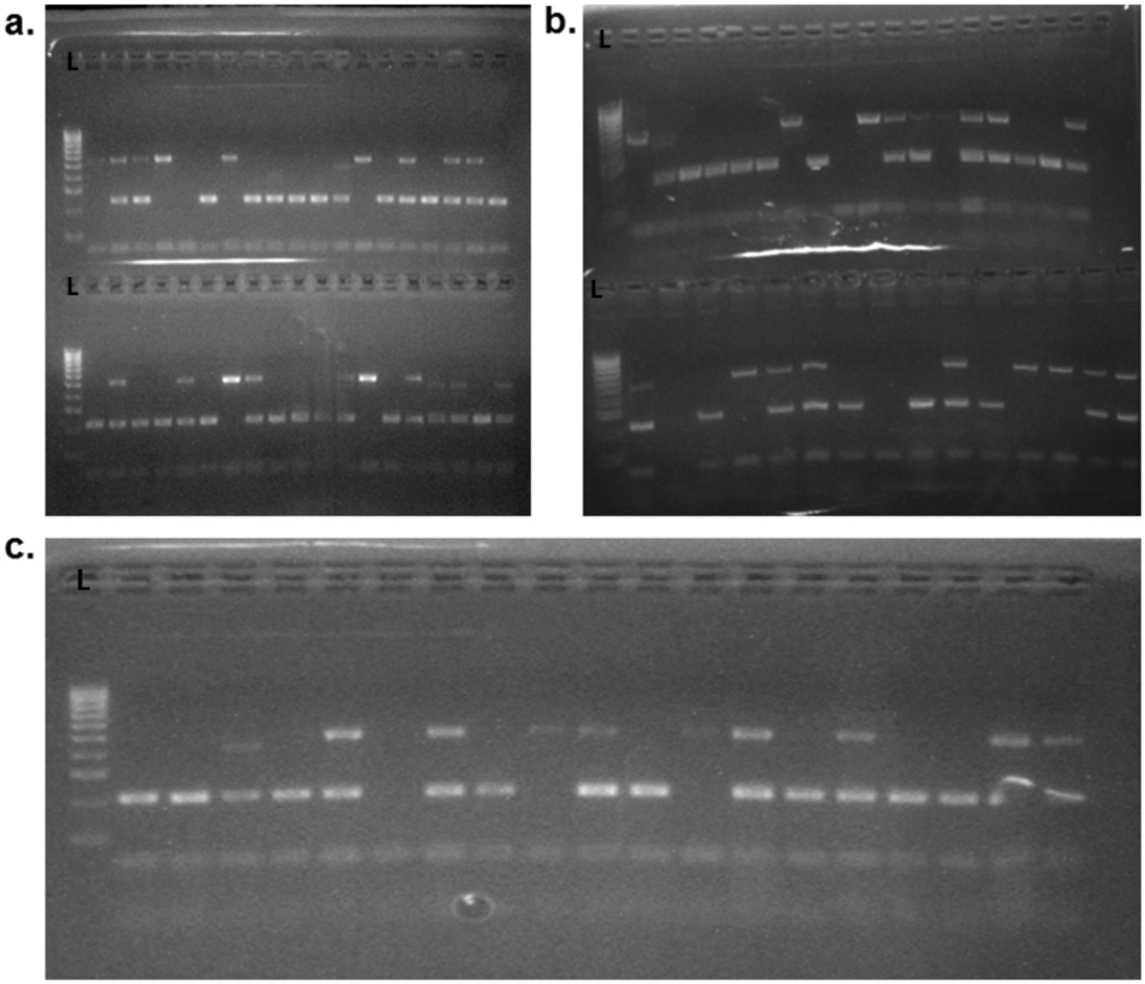

Figure S3. Agarose gel of 2La karyotyping using larvae from permethrin bioassay. **a.** BUK alive (lanes 1–9), BUK dead (10–20), Gamjin Bappa alive (21–26) and Gamjin Bappa dead (27–37); **b.** Pantami alive (1–15) and Pantami dead (16–30); **c.** Auyo alive 1–11 and Auyo dead 12–17. Number 18 not identified from species identification. L is Bioline 100 bp DNA ladder ((100–1013 bp (40–200 ng/band)).

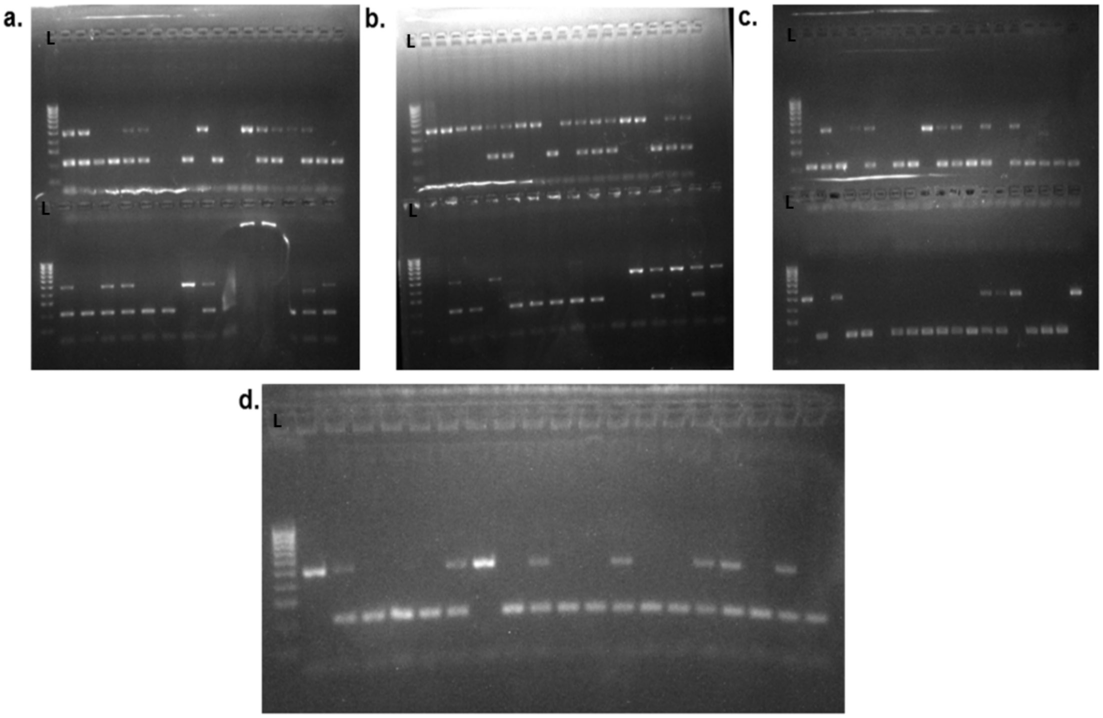

Figure S4. Agarose gel of 2La karyotyping using adult females from permethrin bioassay. **a.** Auyo alive (lanes 1–16), Auyo dead (17-28), BUK alive (29–31); **b.** BUK alive (1–19), BUK dead (20–29), Pantami alive (30); **c.** Pantami alive (1–16), Pantami dead (17–26), Gamjin Bappa dead (27–35); and **d.** Gamjin Bappa alive (1–19). L is Bioline 100 bp DNA ladder ((100–1013 bp (40–200 ng/band)).

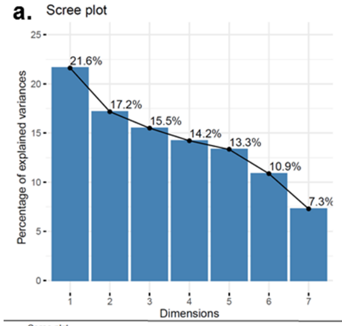

b. Variable cos2

	Dim 1	Dim 2	Dim 3	Dim 4	Dim 5
An. coluzzii	0.67154217	8.539973e-05	0.0111284	0.0001217107	0.082735234
An. gambiae	0.67154217	8.539973e-05	0.0111284	0.0001217107	0.082735234
Auyo	0.05663606	2.363686e-01	0.3172798	0.0859697791	0.113258839
BUK	0.26927530	7.429681e-03	0.2461298	0.3349733287	0.015797401
Gamjin Bappa	0.25090178	1.238897e-01	0.1933297	0.3033460921	0.003448191
Pantami	0.08341684	4.918775e-02	0.1649817	0.5898871777	0.017547515

c. Variable contribution

	Dim 1	Dim 2	Dim 3	Dim 4	Dim 5
An. coluzzii	13.128193	0.002102368	0.3035364	0.003618359	2.6231027
An. gambiae	31.236045	0.005002187	0.7222072	0.008609200	6.2411754
Auyo	2.901618	15.249572129	22.6796332	6.697985735	9.4104956
BUK	12.888089	0.447798678	16.4362411	24.381105435	1.2262267
Gamjin Bappa	13.361785	8.308387044	14.3650036	24.566897325	0.2978141
Pantami	3.823810	2.839358312	10.5517560	41.120906849	1.3045217

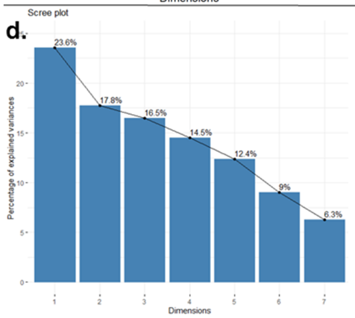

e. Variable cos2

	Dim 1	Dim 2	Dim 3	Dim 4	Dim 5
An. coluzzii	0.58164089	0.136564525	0.001092074	0.004790642	0.032024904
An. gambiae	0.58164089	0.136564525	0.001092074	0.004790642	0.032024904
Auyo	0.11584101	0.002264901	0.498544235	0.005081902	0.295772144
BUK	0.26837029	0.053665060	0.220763028	0.189121139	0.172550212
Gamjin Baffa	0.01786487	0.129259742	0.127137836	0.629680851	0.000743773
Pantami	0.58802236	0.005173522	0.020912506	0.177646754	0.019897681

f. Variable contribution

	Dim 1	Dim 2	Dim 3	Dim 4	Dim 5
An. coluzzii	9.5893208	2.9875212	0.02570753	0.1282319	1.0065236
An. gambiae	25.6746330	7.9988471	0.06882983	0.3433305	2.6948858
Auyo	5.2982446	0.1374545	32.55733214	0.3773682	25.7887424
BUK	11.7036011	3.1053952	13.74633283	13.3904313	14.3451071
Gamjin Baffa	0.8170895	7.8446422	8.30271107	46.7583928	0.0648505
Pantami	27.5199563	0.3212775	1.39744721	13.4983469	1.7752502

Figure S5. Relative contribution of variables on the factor map. **a., b.** and **c.** are Scree plot of the 7 dimensions, cos2 and variables contributions, respectively for dimensions 1–5, from heat stress bioassay. Highest values reflect highest contribution to the dimension (variability); **d., e.** and **f.** are Scree plot of the 7 dimensions, cos2 and variables contributions, respectively for dimensions 1–5, from adult bioassays with permethrin.

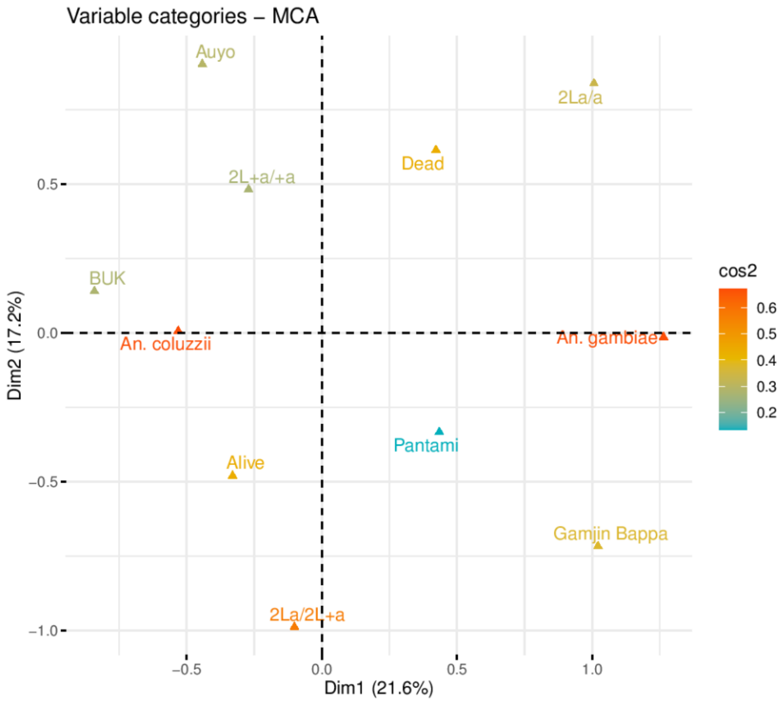

Figure S6. cos2 of variables with *An. coluzzii* and *An. gambiae s.s.* (*An. gambiae* in the plot) exhibiting the highest inertia/contribution.

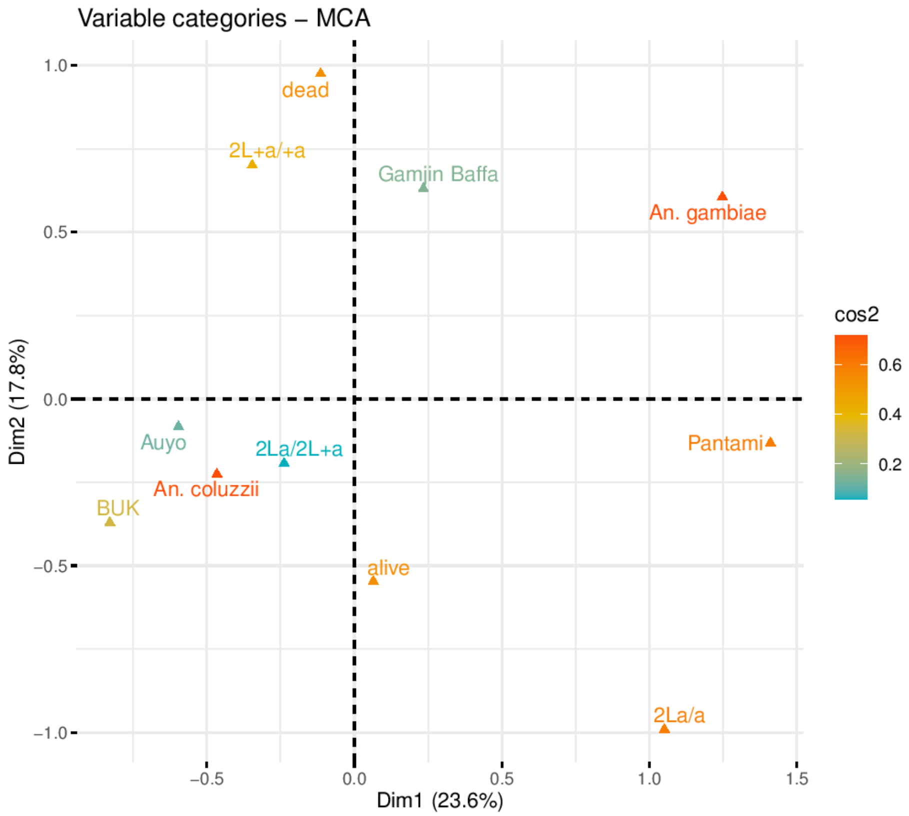

Figure S7. cos2 of the variables with *An. coluzzii* and *An. gambiae s.s.* (*An. gambiae* in the plot) exhibiting the highest inertia/contribution.

Table S1. Primers used for qRT-PCR.

Gene Name	Gene ID	Primer Name	Sequence (5′–3′)	Size (bp)	Efficiency
hsp90	AGAP006959	qhsp90_AGAP006959-F<br>qhsp90_AGAP006959-R	AAGCAGATCGTGAACAAGCA<br>GCCTTCATGATGCGCTCC	227	0.898
hsp90_beta	AGAP001424	qhsp90_AGAP001424-F<br>qhsp90_AGAP001424-R	GCTGTGTGCGGTGGTAATTA<br>CTTGATGGCTTCCTCTTCGC	213	0.946
hsp83	AGAP006958	qhsp83-F<br>qhsp83-R	CCTTCGCATTTCAGGCTGAG<br>GACTCCAGCTTCGAGGGATC	160	0.987
hsp70 1/8	AGAP004944	qhsp70 1/8-F<br>qhsp70 1/8-R	GAGATGCTGCCAAGAACCAG<br>GGCCGTTTCCTTCATCTTGG	239	0.898
hsp90 ATPase	AGAP010514	qhsp_AGAP010514-F<br>qhsp_AGAP010514-R	GGACATGGTGACCGCATTC<br>CCGGAATCATCGTTTGGGTC	239	0.892
hsp70	AGAP004581	qhsp70-F<br>qhsp70-R	CGCAAGTTCAAGAAGGACGT<br>TCGAGCGGAACAGATCAGAG	199	0.974
TPS 1/2	AGAP008227	qTPS1/2-F<br>qTPS1/2-R	CAGGAGGCACAAACCAAGAG<br>AGTTGGTGTGTTGTCCGTTG	237	0.932
IR21a	AGAP008511	qIR21a-F<br>qIR21a-R	GTTGGAGATGCGGCTGTTG<br>AGAATCACCGCTACCCAGAC	300	0.971
IR25a	AGAP010272	qIR25a-F<br>qIR25a-R	GCAATTATAGCGATCATGGGAC<br>AAGTGTGGCGGTCGATTTTC	245	0.817
Elongation factor Tu	AGAP005128	EF-F<br>EF-R	GGCAAGAGGCATAACGATCAATGCG<br>GTCCATCTGCGACGCTCCGG	130	0.979
RPS7	AGAP010592	RPS7-F<br>RPS7-R	CCACCATCGAACACAAAGTTGA<br>TGCTGCAAACTTCGGCTATTC	98	0.897

Table S2. Summary of the 2La inversion distribution in larvae and adult *Anopheles* mosquitoes.

Site / Phenotype	Karyotype			Total
	**Heat stress assay (44 °C for 30 min)**			
	Auyo			
	2La/a	2La/+a	2L+a/+a	
Alive	2	2	9	13
Dead	2	3	4	9
	BUK			
Alive	1	8*	5*	14
Dead	2	2*	9*	13
	Gamjin Bappa 44 °C			
Alive	1	4	7	12
Dead	3	4	0	7
	Pantami			
Alive	2	9	5	16
Dead	3	4	7	14
	**Larval bioassay (100 mg/mL Permethrin)**			
	Auyo			
Alive	2	4	5	11
Dead	0	3	3	6
	BUK			
Alive	3	2	4	9
Dead	1	4	6	11

Gamjin Bappa				
Alive	1	2	3	6
Dead	1	5	5	11
Pantami				
Alive	4	5	6	15
Dead	3	8	5	16
**Adult bioassay (0.75 % Permethrin)**				
Auyo				
Alive	3†	9	3†	15
Dead	1†	5	6†	12
BUK				
Alive	4†	9	9†	22
Dead	0†	2	8†	10
Gamjin Bappa				
Alive	5†	5	9†	19
Dead	0†	4	5†	9
Pantami				
Alive	9†	4	2†	15
Dead	3†	4	3†	10

* statistically significant between alive and dead for $2La/+^a$ vs $2L+^a/+^a$ for BUK, OR = 7.2 (1.08 – 4.79), $\chi^2 = 4.68$, $p = 0.03$.
† statistically significant between alive and dead for 2La/a vs $2L+^a/+^a$ for all sites, OR = 5.02 (1.48 – 6.93), $\chi^2 = 7.45$, $p = 0.01$.

Supplementary Information: Ambient temperature

Monthly averages of ambient temperature for the 4 sampling sites based on a statistical analysis of historical hourly weather reports and model reconstructions from 1 January 1980 to 31 December 2016. The weather-related data were taken entirely from NASA's MERRA-2 satellite-era reanalysis. This reanalysis combines a variety of wide-area measurements in a state-of-the-art global meteorological model to reconstruct the hourly history of weather throughout the world on a 50-kilometer grid (https://weatherspark.com/ accessed on 6 June 2021).

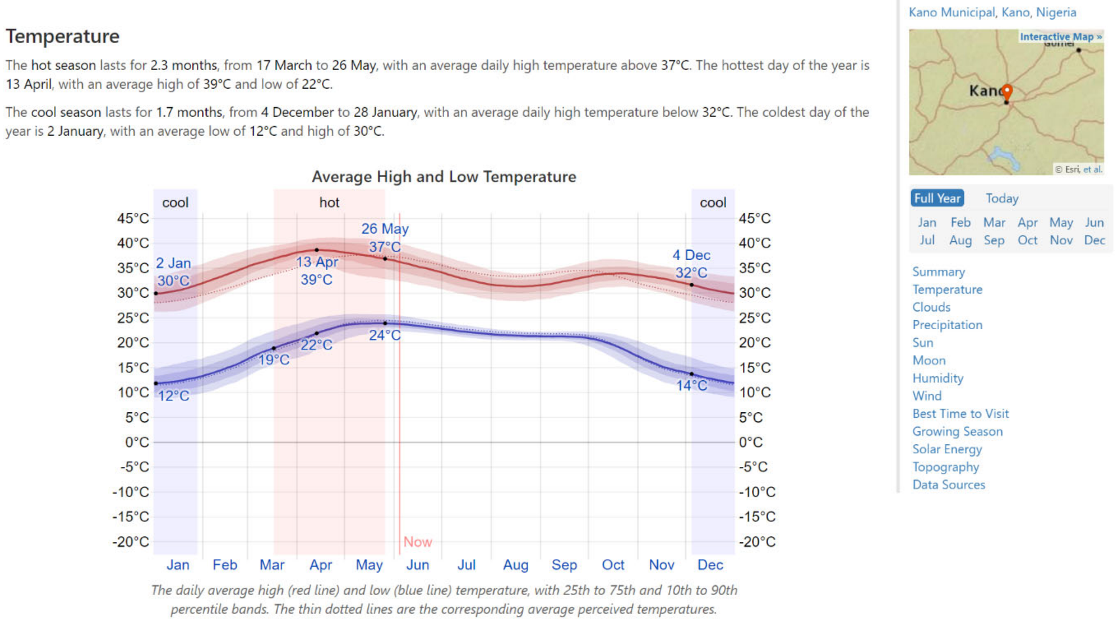

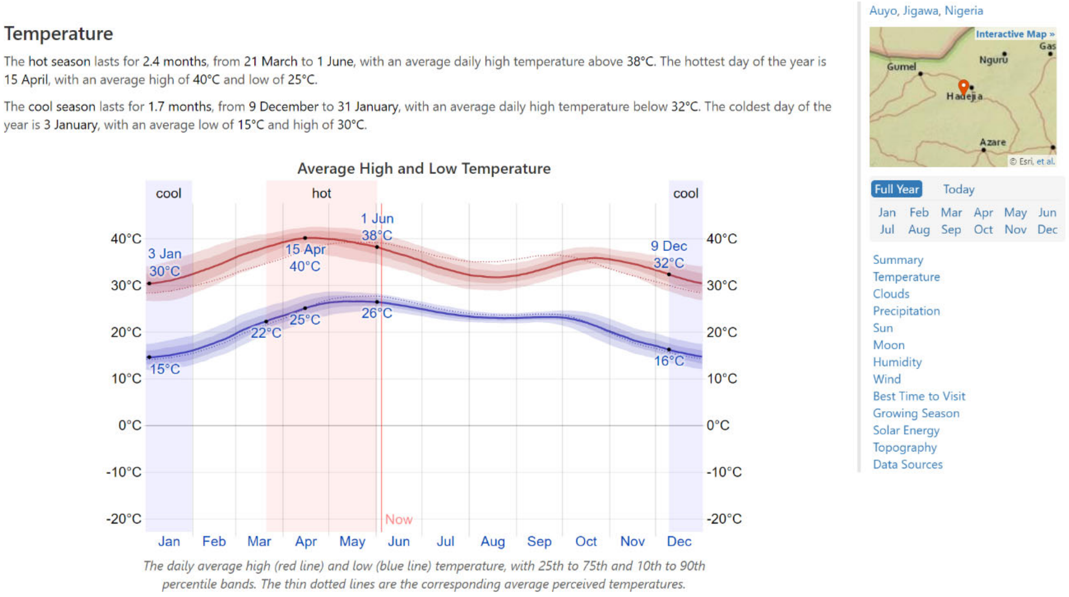
Temperature
The hot season lasts for 2.4 months, from 21 March to 1 June, with an average daily high temperature above 38°C. The hottest day of the year is 15 April, with an average high of 40°C and low of 25°C.
The cool season lasts for 1.7 months, from 9 December to 31 January, with an average daily high temperature below 32°C. The coldest day of the year is 3 January, with an average low of 15°C and high of 30°C.
Average High and Low Temperature
cool
hot
cool
1 Jun 38°C
3 Jan 30°C
15 Apr 40°C
9 Dec 32°C
22°C
25°C
26°C
15°C
16°C
40°C
30°C
20°C
10°C
0°C
-10°C
-20°C
Now
Jan Feb Mar Apr May Jun Jul Aug Sep Oct Nov Dec
The daily average high (red line) and low (blue line) temperature, with 25th to 75th and 10th to 90th percentile bands. The thin dotted lines are the corresponding average perceived temperatures.
Auyo, Jigawa, Nigeria
Interactive Map »
Gumel
Nguru
Hadejia
Azare
© Esri, et al.
Full Year
Today
Jan Feb Mar Apr May Jun
Jul Aug Sep Oct Nov Dec
Summary
Temperature
Clouds
Precipitation
Sun
Moon
Humidity
Wind
Best Time to Visit
Growing Season
Solar Energy
Topography
Data Sources

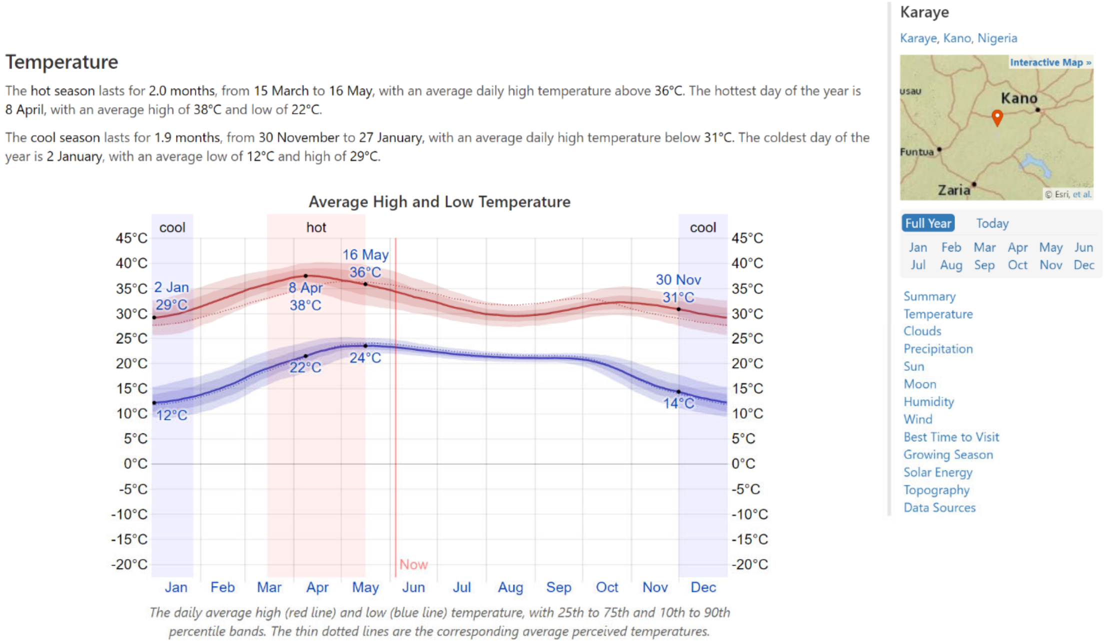
Karaye
Karaye, Kano, Nigeria
Interactive Map »
Kano
Funtua
Zaria
© Esri, et al.
Full Year
Today
Jan Feb Mar Apr May Jun
Jul Aug Sep Oct Nov Dec
Summary
Temperature
Clouds
Precipitation
Sun
Moon
Humidity
Wind
Best Time to Visit
Growing Season
Solar Energy
Topography
Data Sources
Temperature
The hot season lasts for 2.0 months, from 15 March to 16 May, with an average daily high temperature above 36°C. The hottest day of the year is 8 April, with an average high of 38°C and low of 22°C.
The cool season lasts for 1.9 months, from 30 November to 27 January, with an average daily high temperature below 31°C. The coldest day of the year is 2 January, with an average low of 12°C and high of 29°C.
Average High and Low Temperature
cool
hot
cool
16 May 36°C
2 Jan 29°C
8 Apr 38°C
30 Nov 31°C
22°C
24°C
12°C
14°C
45°C
40°C
35°C
30°C
25°C
20°C
15°C
10°C
5°C
0°C
-5°C
-10°C
-15°C
-20°C
Now
Jan Feb Mar Apr May Jun Jul Aug Sep Oct Nov Dec
The daily average high (red line) and low (blue line) temperature, with 25th to 75th and 10th to 90th percentile bands. The thin dotted lines are the corresponding average perceived temperatures.

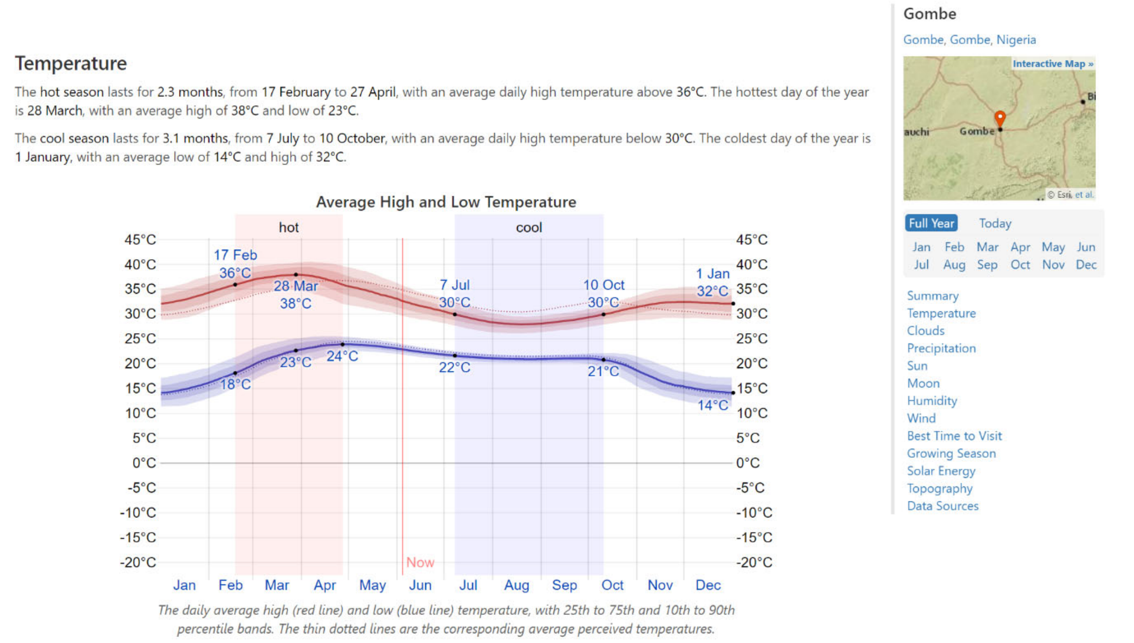

Files S1–S3 are provided separately as Excel files.
